# The evaluation of different types fecal bacteria products for the treatment of recurrent *Clostridium difficile* associated diarrhea: A systematic review and network meta-analysis

**DOI:** 10.3389/fsurg.2022.927970

**Published:** 2022-07-20

**Authors:** Liping Yang, Wenrui Li, Xianzhuo Zhang, Jinhui Tian, Xiaojia Ma, Lulu Han, Huaping Wei, Wenbo Meng

**Affiliations:** ^1^ Department of General Surgery, The First Hospital of Lanzhou University, Lanzhou, China; ^2^ School of Nursing, Lanzhou University, Lanzhou, China; ^3^ The First School of Clinical Medicine, Lanzhou University, Lanzhou, China; ^4^ Evidence-Based Medicine Center, Lanzhou University, Lanzhou, China; ^5^ Lanzhou Library, Chinese Academy of Sciences, Lanzhou, China; ^6^ Department of Nursing, The First Hospital of Lanzhou University, Lanzhou, China

**Keywords:** Fecal microbiota transplantation, clostridium difficile infection, efficacy, safety, network meta-analysis

## Abstract

**Purpose:**

To determine the efficacy of different types of fecal microbiota transplantation for the treatment of recurrent clostridium difficile associated diarrhea (RCDAD).

**Methods:**

We searched PubMed, Embase, The Cochrane Library, Web of Science, China Biomedical Medicine (CBM), China National Knowledge Infrastructure (CNKI) and WanFang database. We also tracked the references found in systematic reviews of RCDAD treated with fecal microbiota transplantation. We included randomized controlled trials (RCTs) comparing different types of fecal microbiota transplantation with other methods for the treatment of RCDAD. The search period was from the date of inception of this treatment method to January 16, 2022. Two reviewers independently screened the published literature, extracted the data and assessed the risk of bias. Systematic review and network meta-analysis were conducted using RevMan 5.4, Stata 16.0 and R 4.1.2 software.

**Results:**

Ten RCTs involving 765 patients were included in this network meta-analysis. The results showed that treatment with fresh fecal bacteria and frozen fecal bacteria were better than vancomycin, fresh vs. vancomycin [odds ratio, (OR) = 8.98, 95% confidence interval (95% CI) (1.84, 43.92)], frozen vs. vancomycin [OR = 7.44, 95% CI (1.39, 39.75)]. However, there were no statistically significant differences in cure rate [fresh vs. frozen: OR = 1.21, 95% CI (0.22, 6.77); fresh vs. lyophilized, OR = 1.95, 95% CI (0.20, 19.44); frozen vs. lyophilized, OR = 1.62, 95% CI (0.30, 8.85)]. The Surface Under the Cumulative Ranking (SUCRA) indicated that fresh fecal bacteria were the best treatment for RCDAD.

**Conclusions:**

Fresh fecal bacteria are the best treatment of RCDAD, frozen fecal bacteria and lyophilized fecal bacteria can achieve the same effect. Fecal microbiota transplantation is worthy of clinical and commercial application.

## Introduction

Clostridium difficile (CD) is gram-positive anaerobic bacteria that was originally reported by Hall and O'Toole in 1935 as a component of the fecal flora of healthy newborn infants (1). It is widely distributed in the natural environment, animal and human feces, and belongs to the normal intestinal flora. CD infection (CDI) is the main cause of diarrhea in hospitals, accounting for 20% to 30% of all antibiotic-related cases (2). Age, comorbidities and the use of antibiotics are the main risk factors (3). The incidence of CDI in hospitals and communities is increasing, posing a serious challenge for public health (4–7). The latest data showed that nearly 20% of patients were diagnosed with CDI after receiving standard antibiotic therapy, and the recurrence rate was as high as 50% to 60% (8, 9). Due to its resistance to antibiotics, recurrent CDI (rCDI) is more likely to produce serious clinical manifestations, such as inflammatory lesions and the formation of pseudo-membranes, which increase the risk of life-threatening complications (toxic megacolon, sepsis) and death (10). Fecal microbiota transplantation (FMT) is an effective method for treating recurrent or refractory CDI (11), since FMT can restore the diversity and function of the intestinal flora, allowing it to resist CD and its toxins (12, 13). In recent years, the FMT has been commonly used in clinical practice and recommended for treating multiple recurrences of CDI in international guidelines (14). However, there is a lack of evidence of evidence-based medicine comparing the efficacy of fresh fecal bacteria, frozen fecal bacteria, lyophilized fecal bacteria and the autologous fecal bacteria for the treatment of rCDI. Hence, the advantages and disadvantages of different forms of FMT remain questionable. Therefore, it is necessary to evaluate the efficacy of different forms of FMT for treating rCDI.

With improvements in theoretical systems and methods, new meta-analyses are constantly being conducted (15). Based on the traditional meta-analysis, network meta-analysis (NMA) was developed, making it possible to simultaneously compare multiple interventions. The main purpose of NMA is to comprehensively evaluate and rank all interventions at the same time (16). Towards this goal, we performed a systematic review and NMA comparing the effectiveness of FMTs and provide scientifically reliable evidence of the effectiveness of FMT in clinical practice.

## Methods

### Study design

This systematic review and network meta-analysis were reported in accordance with the Preferred Reporting Items for Systematic Reviews and Meta-Analyses (PRISMA) for Network Meta-Analyses (PRISMA-NMA) reporting standard (17), and were registered in the International Prospective Register of Systematic Reviews (PROSPERO: CRD42020150064) (18).

### Selection criteria

We included studies based on the following criteria: (1) Study participants ≥18 years with rCDI; (2) Interventions: comparison between FMT and FMT or antibiotics. FMT mainly included: fresh fecal bacteria, frozen fecal bacteria, lyophilized fecal bacteria, and autologous fecal bacteria; (3) Study design: randomized controlled trial (RCT); (4) Outcomes: cure rate (clinical cure was defined as lack of CDI recurrence with maintenance of resolution (that is, <3 unformed stools per day) for 8 weeks without requirement for further antibiotics (metronidazole, vancomycin, or fidaxomicin).

We excluded studies based on the following criteria: (1) Non-Chinese and non-English language studies; (2) Republished studies; (3) Studies of FMT combining a variety of treatments; (4) Retrospective and historical comparison studies.

### Search strategy

We systematically searched PubMed, Cochrane Library, Web of Science, Embase, China Biomedical Medicine (CBM), China National Knowledge Infrastructure (CNKI), and WanFang databases. The search period was from the date of inception to January 16, 2022. The search strategy involved multiple pre-retrievals, and the language was unlimited. We also tracked relevant reviews and systematic reviews/meta-analyses. In addition, search engines such as Google were used to retrieve relevant studies and grey literature on the Internet. We also tracked referenced studies as a supplementary search. We conducted the search using a combination of subject and free words. The main search terms used in English language databases were the following: “fecal”, “faecal”, “microbiota”, “feces”, “faeces”, “stool”, “fecal flora”, “faecal flora”, “transplant”, “transfusion”, “implantation”, “implant”, “instillation”, “microbiota”, “donor”, “enema”, “reconstitution”, “infusion”, “therapy”, “bacteriotherapy”, “clostridium difficile”, “infection”, “CDI”, “randomized controlled trial”, “RCT”. Two reviewers independently conducted the search.

### Literature selection and data extraction

Search records were imported into EndNote X9 literature management software. Two reviewers independently reviewed the titles and abstracts of the studies based on the inclusion and exclusion criteria. Next, the full texts of the selected studies were read and the data extracted. Dissenting points of view were discussed to reach a consensus. Two reviewers independently extracted data using a pre-designed Excel sheet which reviewers had been previously trained to use. The items extracted included (title, author, year of publication, country), participants' characteristics (sample size, average age, gender, fecal type, infusion pathway and volume, details of the intervention, outcomes, and measured results).

### Risk of bias in individual studies

After training, two authors independently assessed the risk of bias of the included RCTs based on the Cochrane Handbook Version 5.1.0 (19), and the following items were reported: random sequence generation, allocation concealment, blinding of participants and personnel, blinding of outcome assessment, incomplete outcome data, selective reporting and other bias. These items were evaluated as showing high, low or unclear risk of bias. Any disagreements were resolved through discussion and by reaching a consensus with a third reviewer.

### Statistical analysis

We drew a network diagram using the “network plot” command of the Stata (V.16.0) program to ensure that the included studies form a connected network for each outcome. Standardized Meta-analysis were conducted using RevMan 5.4 software. Bayesian NMA was performed using the Markov Chain Monte Carlo (MCMC) method in the R (V.4.1.2) software package. The probability of each intervention becoming the best was analyzed based on the Surface Under the Cumulative Ranking (SUCRA) probabilities. Meanwhile, we calculated the ranking results for each intervention and assessed the possibility of publication bias by funnel plot analysis (Supplementary Appendix 1).

## Results

### Study selection

**Figure 1:**
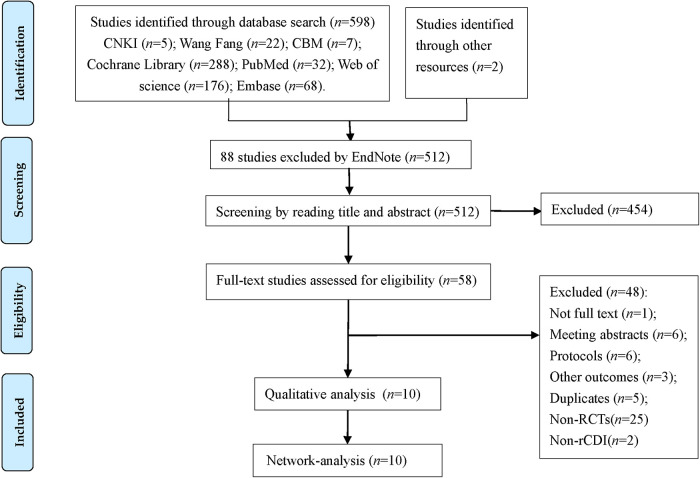
The flow chart of literature searching and screening process.

We identified 598 studies according to the pre-designed search strategy, including 34 studies in Chinese, 564 articles in English, and 2 studies obtained through other pathways. With the help of EndNote X9 software, we removed 88 duplicate studies, excluded 454 studies based on the title and abstract, and then screened the full texts of 58 studies. Finally, 10 RCTs were included in the study. The ﬂow diagram (Figure 1) shows the search results and selection details.

### Characteristics of the included studies

Ten RCTs involving 765 patients were included in the study (20–29). All patients were diagnosed with rCDI and were from Denmark, France, the United States, Canada, Roman, and Netherlands, age ≥18 years old. Seven types of interventions were assessed in the treatment of recurrent clostridium difficile associated diarrhea (RCDAD). The included RCTs focused on 2013–2021 and were all published in the English language. FMT infusion routes include nasal intestinal tube, colonoscopy, enema, oral and nasal duodenum tube. The volume of infusion ranged from 50 g to 200 g. Nine studies (90%) (20, 22–29) compared fresh fecal bacteria, frozen fecal bacteria, lyophilized fecal bacteria, vancomycin, fidaxomicin and rectal bacteriotherapy. Only 1 study (10%) (21) compared frozen fecal bacteria with autologous fecal bacteria. In three of ten studies (30%), participants were randomly assigned to 3 groups. The basic characteristics of the 10 RCTs and clinical characteristics of patients are shown in Table 1.

**Table 1:** Characteristics of the included studies.

Study	Disease	Country	Sample	Sample		Age (years)		Gender(male-female)		Intervention		Fecal type	FMT infusion pathway	Infusion volume	Outcomes
				T	C	T	C	T	C	T	C				
Hvas 2019 (1) (20)	rCDI	Denmark	48	24	24	22–90	24–87	4–20	11–13	B	F	B	Nasal intestinal tube or colonoscopy	50 g	①
Hvas2019(2) (20)	rCDI	Denmark	40	24	16	22–90	21–92	4–20	5–11	B	E	B	Nasal intestinal tube or colonoscopy	50 g	①
Kelly 2016 (21)	rCDI	France, America	46	22	24	48 ± 16	55 ± 14	4–18	5–19	B	D	B,D	Colonoscopy	100 g	①
Lee 2016 (22)	rCDI	Canada	219	111	108	72.5 ± 16.2	73.0 ± 16.4	37–74	36–72	A	B	A,B	Enema	64 g	①
kao 2017 (23)	rCDI	Canada	105	52	53	57.4 ± 19.1	58.7 ± 18.5	39%–61%	24.6%–75.4%	B	C	B,C	Colonoscopy, oral	360 ml, 40capsules	①
Jiang 2017 (1) (24)	rCDI	America	49	25	24	19–97	33–88	4–21	6–18	A	B	A,B	Colonoscopy	50 g	①
Jiang 2017 (2) (24)	rCDI	America	48	25	23	19–97	20–87	4–21	10–13	A	C	A,C	Colonoscopy	50 g	①
Jiang 2017 (3) (24)	rCDI	America	47	24	23	33–88	20–87	6–18	10–13	B	C	B,C	Colonoscopy	50 g	①
Jiang 2018 (25)	rCDI	America	65	34	31	63	67	9–25	10–21	B	C	B,C	Enema, oral	100–200 g/100 g	①
Hota 2017 (26)	rCDI	Canada	28	16	12	75.7 ± 14.5	69.6 ± 14.2	5–11	4–8	A	E	A	Enema	50 g	①
Cammarota 2015 (27)	rCDI	Roman	39	20	19	71 ± 15	75 ± 11	8–12	8–11	A	E	A	Colonoscopy	152 ± 32 g	①
van Nood 2013 (28)	rCDI	Netherlands	29	16	13	73 ± 13	66 ± 14	8–8	5–7	A	E	A	Nasal-duodenum tube	141 ± 71 g	①
Rode AA 2021(1)(29)	rCDI	Denmark	65	34	31	75(66–81)	76(65–84)	14–20	17–14	B	E	B	Enema	50 g	①
Rode AA 2021 (2) (29)	rCDI	Denmark	67	34	33	75(66–81	67(60–79)	14–20	14–19	B	G	B	Enema	50 g	①

*RCDI, Recurrent clostridium difficile infection; T, Treatment group; C, Control group; ① Cure rate.*

*(**A**) fresh fecal bacteria; (**B**) frozen fecal bacteria; (**C**) lyophilized fecal bacteria; (**D**) autologous fecal bacteria; (**E**) vancomycin; (**F**) fidaxomicin; (**G**) rectal bacteriotherapy.*

### Methodological quality of the included studies

**Figure 2:**
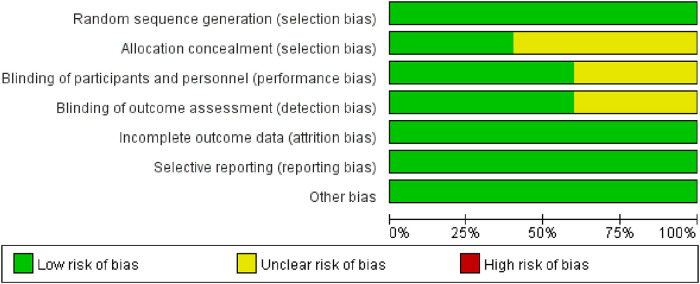
Risk of bias in included studies.

Of the 10 RCTs, two studies (20%) (24, 25) were A-level, and the rest (20–23, 26–29) were B-level. Five studies (50%) (22–25, 27) used a computer-generated random number list for random sequence generation, and four studies (24, 25, 27, 29) used allocation concealment. Six studies (60%) (21, 22, 24, 25, 28, 29) reported the use of blinding methods for investigators and patients. We evaluated the “loss to follow-up” from the number of grouped cases and the number of results reports. Ten RCTs (100%) had no missing data. The quality evaluation showed that potential bias was caused by inadequate random sequence generation and allocation concealment, as well as by a lack of blinding of participants and personnel (Figure 2).

### Standardized meta-analysis

**Figure 3:**
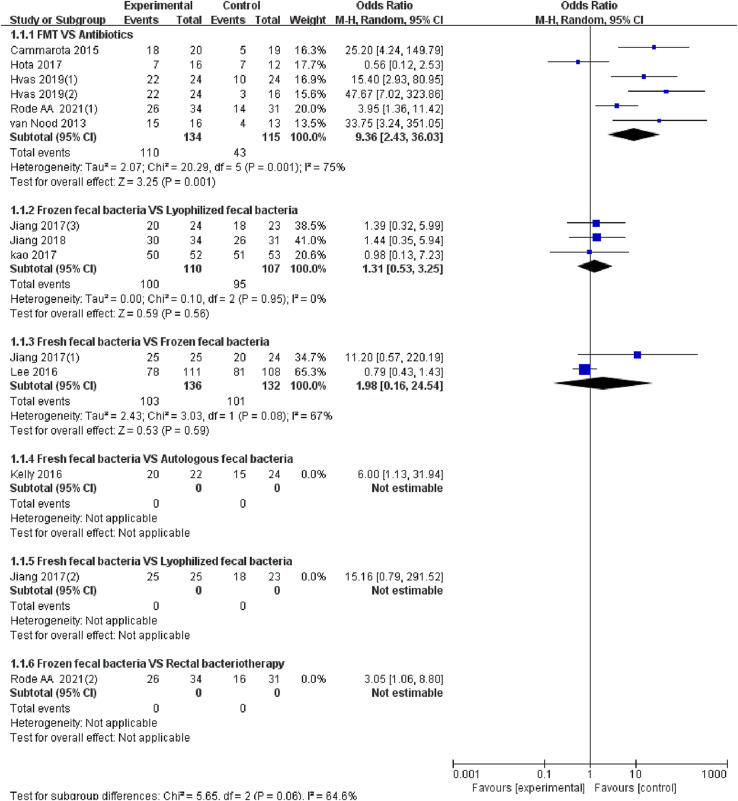
Forest of cure rate of different FMT for rCDI associated diarrhea.

The 10 studies (20–29) reported the cure of RCDAD. The results of the heterogeneity test (*I*^2^ > 50%, *p *< 0.05), the random effect model was used for meta-analysis. The results of subgroup analysis showed that the FMT was significantly better than antibiotic treatment in the cure rate of RCDAD (OR = 9.36, 95% CI: 2.43–36.03, *p *= 0.001) (82.1% vs. 37.4%), but the comparison between frozen fecal bacteria and lyophilized fecal bacteria (OR = 1.31, 95% CI: 0.53–3.25, *p* = 0.95) (90.1% vs. 88.8%), fresh fecal bacteria and frozen fecal bacteria (OR = 1.98, 95% CI: 0.16–24.54, *p* = 0.08) (75.7% vs. 76.5%) did not reach a significant difference (*p* > 0.05) (Figure 3).

### Results of the network meta-analysis

#### The network plots of different FMT

**Figure 4:**
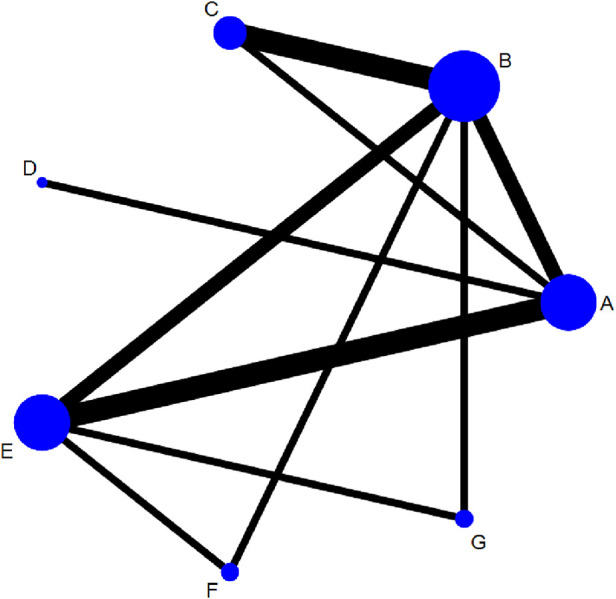
Networks for multiple treatment comparisons of cure rate. Lines between points indicate evidence of direct comparisons between two interventions. The thickness of lines indicates the number of studies using the two treatments, whereas the size of the dots indicates the total sample size of the intervention. (**A**) fresh fecal bacteria; (**B**) frozen fecal bacteria; (**C**) lyophilized fecal bacteria; (**D**) autologous fecal bacteria; (**E**) vancomycin; (**F**) fidaxomicin; (**G**) rectal bacteriotherapy.

Figure 4 shows the network structure of the comparisons among different interventions for the outcomes. Nodes represent different interventions and the lines between the intervention nodes indicate the direct comparisons made within RCTs. The thickness of the edge reflects the number of included trials, and is proportional to the number of trials comparing each pair of interventions. The size of the node reflects the sample size of the intervention, and it is proportional to the number of randomly assigned participants (e.g., the sample size). The closed loop shows that there are both direct and indirect comparisons, and missing links between interventions reflect the lack of direct comparisons.

#### Network analysis

The NMA showed that fresh fecal bacteria and frozen fecal bacteria were superior to vancomycin to treat RCDAD, and the difference was statistically significant [fresh fecal bacteria vs. vancomycin (OR = 8.98, 95% CI 1.84–43.92), frozen fecal bacteria vs. vancomycin (OR = 7.44, 95% CI 1.39–39.75)]. However, differences between FMT modalities (fresh, frozen, lyophilized or autologous fecal bacteria were not statistically significant. The NMA results are shown in Table 2.

**Table 2:** Head-to-head comparisons of efficacy of FMT.

A	0.83(0.15,4.64)	0.51(0.05,5.09)	0.17 (0.01,3.51)	0.11(0.02,0.5)	0.14(0.01,2.64)	0.20 (0.01,3.15)
1.21 (0.22,6.77)	B	0.62 (0.11,3.38)	0.20 (0.01,6.67)	0.13(0.03,0.72)	0.17 (0.01,2.53)	0.24 (0.02,2.97)
1.95 (0.20,19.44)	1.62 (0.30,8.85)	C	0.33 (0.01,14.79)	0.22 (0.02,2.19)	0.28 (0.01,6.49)	0.38 (0.02,7.83)
6.00 (0.28,126.34)	4.97(0.15,164.69)	3.07 (0.07,139.57)	D	0.67(0.02,20.74)	0.86(0.01,58.13)	1.18 (0.02,72.65)
8.98 (1.84,43.92)	7.44(1.39,39.75)	4.60 (0.46,46.37)	1.50 (0.05,46.49)	E	1.28(0.09,18.32)	1.77(0.14,21.87)
7.01 (0.38,129.38)	5.80(0.40,85.13)	3.59 (0.15,83.50)	1.17 (0.02,79.25)	0.78(0.05,11.14)	F	1.38 (0.04,44.62)
5.08 (0.32,81.14)	4.21(0.34,52.48)	2.60 (0.13,52.91)	0.85 (0.01,52.04)	0.57 (0.05,6.99)	0.72(0.02,23.45)	G

*(A) fresh fecal bacteria; (B) frozen fecal bacteria; (C) lyophilized fecal bacteria; (D) autologous fecal bacteria; (E) vancomycin; (F) fidaxomicin; (G) rectal bacteriotherapy.*

#### Rank probabilities

**Figure 5:**
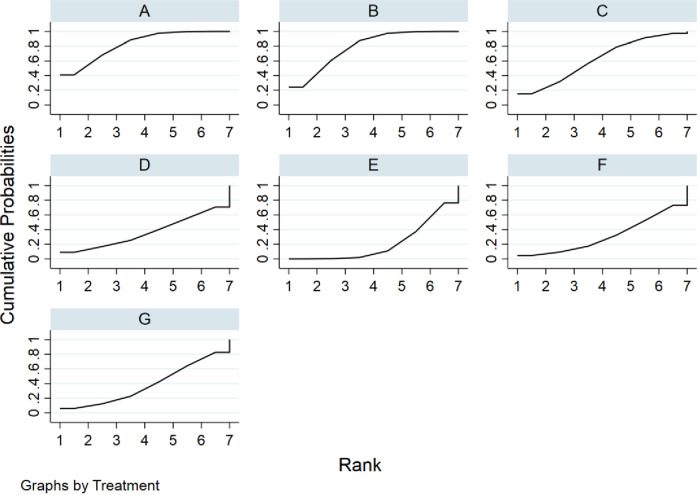
Rank probabilities for cure rate. (**A**) fresh fecal bacteria; (**B**) frozen fecal bacteria; (**C**) lyophilized fecal bacteria; (**D**) autologous fecal bacteria; (**E**) vancomycin; (**F**) fidaxomicin; (**G**) rectal bacteriotherapy.

The SUCRA metric was used to rank the effectiveness of each treatment and identify the best treatment. The SUCRA line shows the percent of effectiveness of each treatment accounting for all possible rankings and uncertainties in treatment effects. SUCRA values range from 1, being the best without uncertainty, to 0, being the worst without uncertainty. The results of the SUCRA show probability ranking in descending order is identified as fresh fecal bacteria, frozen fecal bacteria, lyophilized fecal bacteria, rectal bacteriotherapy, autologous fecal bacteria fidaxomicin and vancomycin (Figure 5).

### Publication bias

**Figure 6:**
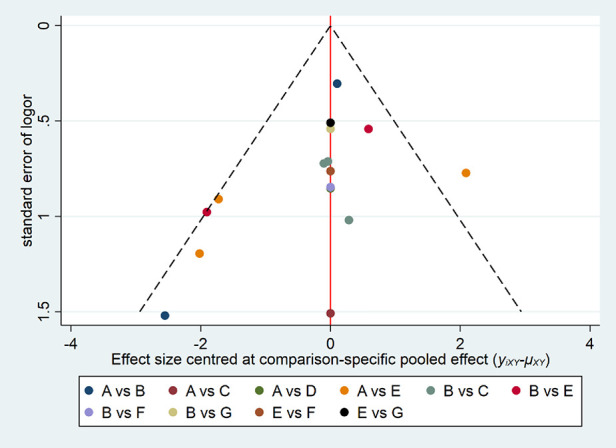
Funnel plot of the cure rate with different forms of FMT for rCDI-associated diarrhea. (**A**) fresh fecal bacteria; (**B**) frozen fecal bacteria; (**C**) lyophilized fecal bacteria; (**D**) autologous fecal bacteria; (**E**) vancomycin; (**F**) fidaxomicin; (**G**) rectal bacteriotherapy.

Comparison-adjusted funnel plots were created for all outcomes (Figure 6). Different colors refer to different comparisons. All studies were symmetrically distributed around the *X *= 0 vertical line, so it can be assumed that included studies were less likely to show publication bias.

## Discussion

FMT, in which the fecal microbiome of a healthy donor is transplanted into a patient, aims to restore the normal gut microbiome and is already a successful therapy for rCDI (30). However, the underlying mechanisms remain unclear. Bacilli and thick-walled bacteria are key components of FMT (31). Mullish et al. (32) reported that FMT accelerated the hydrolysis of taurocholic acid by restoring the activity of bile salt hydrolase in the gut microbiome (33). Although there are still many challenges in FMT, this method has shown therapeutic potential to treat refractory or rCDI (34). We conducted the first network meta-analysis to date on the treatment of recurrence of CDI compared different types of FMT with standard-of-care treatment with antibiotics, and compared with rectal bacterial therapy.

The 10 studies included in this NMA met quality evaluation standards: 2 studies were assessed as being A-level, and 8 studies were B-level. The risk of bias depended mainly on the blinding methods and other biases. The cure rate is an objective outcome. Therefore, the use of blinding methods in these studies brought less bias. Other biases stemmed mainly from unreported information about funding and conflicts of interest. Therefore, the methodological quality of the studies included in this NMA was high and it is hoped that follow-up research will further improve random sequence generation, allocation concealment, blinding methods and data integrity.

The risk of recurrence after antibiotic treatment of CDI has attracted the attention of medical experts, and a high mortality has been reported (35–37). Therefore, CDI remains a significant medical challenge. The meta-analyses (38–40) confirmed that FMT was an effective, safe and economical method to treat rCDI. Unfortunately, there was no indirect comparison of different FMT modalities. Hui's study (41) suggested that fresh fecal bacteria worked better than antibiotics and placebo for rCDI, but the effect of an infusion of fresh fecal bacteria by colonoscopy or enema was not significantly different from that of frozen fecal bacteria or lyophilized fecal bacteria administered through oral capsules. This was consistent with the results of this study.

This NMA confirmed that there were no statistically significant differences between fresh fecal bacteria, frozen fecal bacteria or lyophilized fecal bacteria for the treatment of RCDAD. The reason may be that the number and type of fecal bacteria found in fresh, frozen or lyophilized fecal bacteria preparations are similar (42), so there were no significant differences in terms of therapeutic effects. Since it is difficult to collect fresh fecal bacteria, they can be replaced with frozen fecal bacteria or lyophilized fecal bacteria to treat rCDI in the future. Lyophilized fecal bacteria are easy to store and very useful for patients and doctors, it can be used at any time and have commercial value (43, 44). Lyophilized fecal bacteria not only improve the effectiveness of rCDI treatment, but also provide alternative treatments for rCDI patients. Furthermore, lyophilized fecal bacteria has the potential of large- scale production with a larger capacity than fresh fecal bacteria and frozen fecal bacteria, even when donor stool banks are established.

During the course of FMT treatment, different degrees of bloating, abdominal pain, diarrhea and other manifestations may appear, which are caused by changes in the composition of the gut microbiome, gene expression by mucosal cells, immunologic function of the intestinal mucosa, intestinal ecological environment and differences in body metabolism (45–47). Tang's (48) meta-analysis indicated that FMT was safe for rCDI. Although some serious adverse reactions related to FMT have been reported, these are not serious and do not cause harm to patients. Ten studies described the adverse events, but did not elaborate on the preventive measures. It is hoped that the adverse events produced by FMT for the treatment of rCDI can be studied in detail in the future. Moreover, the finding of a potential reduction in all causes mortality after FMT were reported in two study included in our NMA.

Our study has several limitations. First, current studies have used FMT for the treatment of RCDAD as an example to validate the NMA method, based on the OR value and 95% CI in Stata 16.0 and R 4.1.2 software. However, this method has some limitations and can't comprehensively reflect all the therapeutic effects. To determine the OR value at different time points, NMAs based on the cure rate should be adopted. SUCRA provides an opportunity to determine the best available treatment, one must interpret with caution as high values may only provide supportively, but not conclusive, evidence for treatment options. In addition, this study only focused on the cure rate. The total effective rate, and adverse events rate after FMT for rCDI need to be further analyzed to strengthen the evidence.

## Conclusions

Fresh fecal bacteria and frozen fecal bacteria were superior to vancomycin for the treatment of RCDAD, but there were no significant differences in cure rate between fresh fecal bacteria, frozen fecal bacteria or lyophilized fecal bacteria. Based on the SUCRA analysis, fresh fecal bacteria were the best treatment for RCDAD diarrhea, frozen fecal bacteria and lyophilized fecal bacteria may also achieve the same effect.

## Data Availability

The original contributions presented in the study are included in the article/**Suplementary Material**, further inquiries can be directed to the corresponding author/s.
