# Supplementary material for: The evaluation of different types fecal bacteria products for the treatment of recurrent Clostridium difficile associated diarrhea: A systematic review and network meta-analysis
Source: Front Surg. 2022 Jul 20;9:927970. doi: 10.3389/fsurg.2022.927970 (PMC9709817; doi:10.3389/fsurg.2022.927970)
Supplement: Supplementary file 1 [file Datasheet1.docx]

**Appendix 1**

**Network meta-analysis was performed using R 4.1.2 software.**

**1. Statistical analysis**

The initial value setting was determined by using two Markov chains. The number of initial update iterations of the model was set to 10,000. The number of continuation update iterations was set to 100,000, and the first 10,000 anneals were used to eliminate the initial value effect to begin sampling after 10,001. Heterogeneity was assessed by using the *I*^2^ index. When *I*^2^ was *>*50%, heterogeneity was present between studies and a multi-regression model was used for analysis. Heterogeneity between studies was considered better when *I*^2^ was *≤*50%. The node-split method was used to explore consistency. We considered there was inconsistency between direct and indirect evidence when *P<*0.05. We evaluated the convergence of this NMA using potential scale reduction factor (PSRF); when the PSRF value was close to or equal to 1, it showed that the convergence effect was good. The consistency model analysis conclusion is more credible, and instead the credibility is lower. The probability of each intervention becoming the best was analyzed based on the Rank probabilities.

**2. Results**

**2.1 Convergence assessment**

As shown in the convergence diagnostic diagrams (Figure1), the PSRF value was close to 1. The median value of the reduction factor was 97.5%. It tended to be 1 and achieved stability. The convergence effect was good after 100,000 iterations, showing that the credibility of the consistency model analysis in this NMA was high.


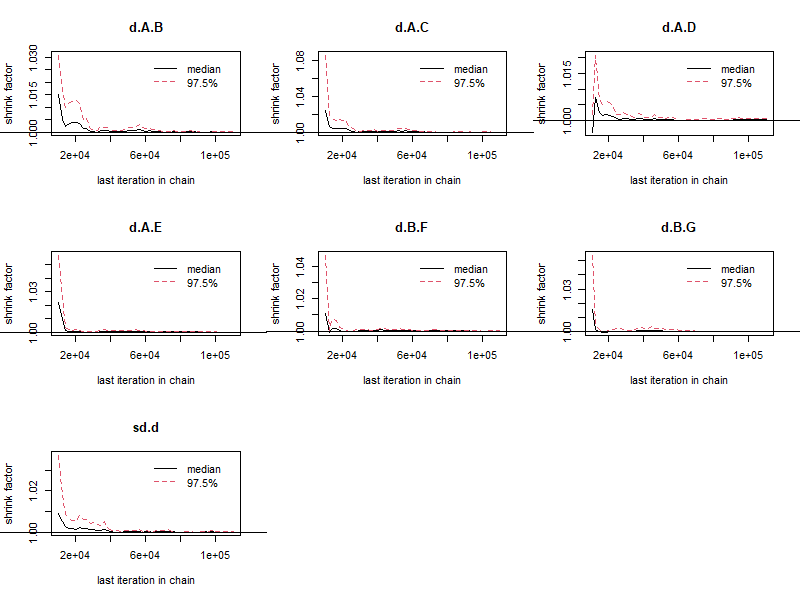


Figure 1. Convergence of cure rate using different FMTs for rCDI-associated diarrhea

A: fresh fecal bacteria; B: frozen fecal bacteria; C: lyophilized fecal bacteria; D: autologous fecal bacteria; E: vancomycin; F: fidaxomicin; G: rectal bacteriotherapy

**2.2 Consistency test**

The results of the node split model showed that the results from direct and indirect comparison was different only when comparing fresh fecal bacteria and lyophilized fecal bacteria (*P*=0.0168), this inconsistency was mainly caused by a different pathway of FMT infusion. Other node split results were consistent, indicating that the consistency of the recovery rate was good enough (*P*>0.05) (Figure 2).


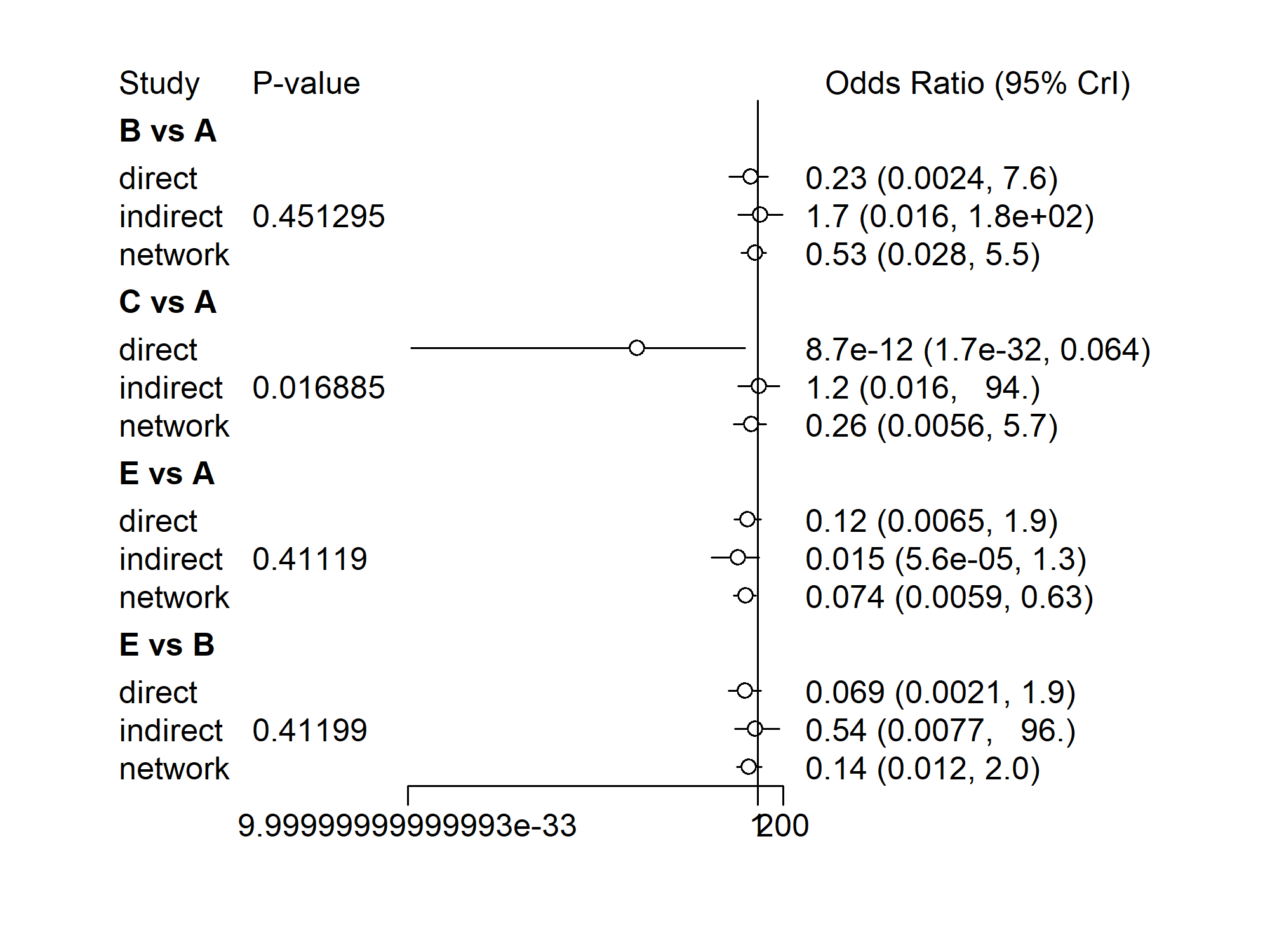


Figure 2 Side-splitting resultscure rate for rCDI-associated diarrhea using different FMTs

A: fresh fecal bacteria; B: frozen fecal bacteria; C: lyophilized fecal bacteria; D: autologous fecal bacteria; E: vancomycin; F: fidaxomicin; G: rectal bacteriotherapy

**2.3 Rank probabilities**

Rankings indicated that the probability of the methods was the best treatment. As shown in Figure 9, Fresh fecal bacteria is the best treatment.

**
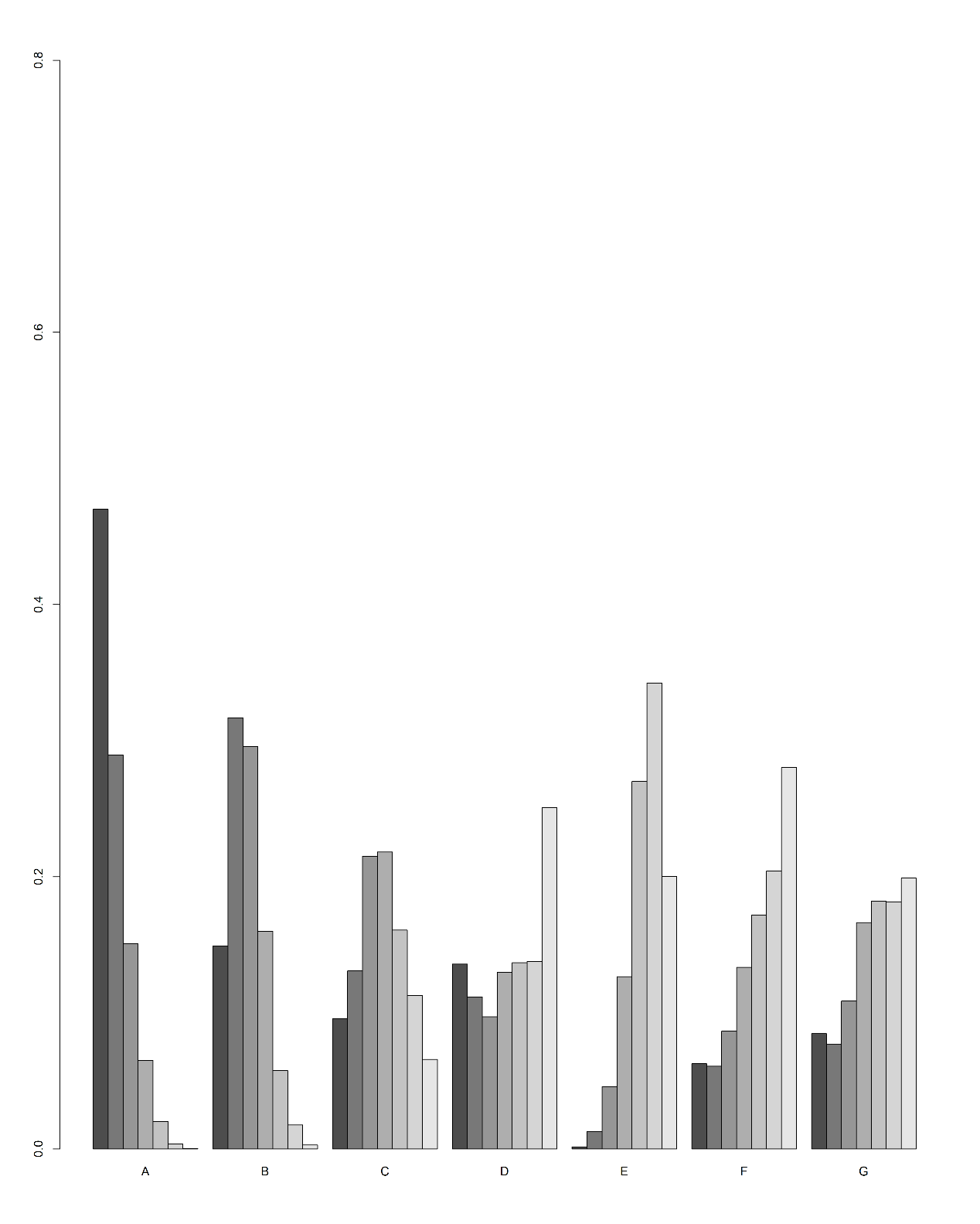
**

Figure 3. Rank probabilities for recovery rate

A: fresh fecal bacteria; B: frozen fecal bacteria; C: lyophilized fecal bacteria; D: autologous fecal bacteria; E: vancomycin; F: fidaxomicin; G: rectal bacteriotherapy

**2.4 Bayesian network analysis**

The NMA showed that fresh fecal bacteria were superior to vancomycin to treat rCDI-associated diarrhea, and the difference was statistically significant (OR = 13.66, 95% CI 1.51 to 167.61). However, differences between FMT modalities (fresh, frozen, lyophilized or autologous fecal bacteria) were not statistically significant. The NMA results are shown in Table 1.

Table 1. Head-to-head comparisons of efficacy of FMT

| A | 0.52(0.03,5.63) | 0.26(0.01,5.57) | 0.14(0, 13.25) | 0.07(0.01,0.66) | 0.09 (0, 5.87) | 0.12 (0, 8.13) |
| --- | --- | --- | --- | --- | --- | --- |
| 1.91 (0.18, 35.78) | B | 0.5 (0.04, 6.19) | 0.27(0, 68.05) | 0.14 (0.01, 2.1) | 0.17(0, 10.46) | 0.24(0, 14.36) |
| 3.78 (0.18, 166.88) | 2 (0.16, 28.2) | C | 0.53(0, 230.45) | 0.28(0.01,10.45) | 0.33 (0, 44.02) | 0.48(0, 61.55) |
| 7.14 (0.08, 793.38) | 3.73(0.01,605.42) | 1.88(0, 434.82) | D | 0.52 (0, 86.11) | 0.62 (0, 322.89) | 0.88(0,453.73) |
| 13.66(1.51,167.61) | 7.11 (0.48, 84.81) | 3.57(0.1,101.31) | 1.91(0.01,368.63) | E | 1.17(0.02,66.06) | 1.7 (0.03, 95.07) |
| 11.62(0.17,1308.75) | 6.03 (0.1, 364.21) | 3.01(0.02,337.55) | 1.62 (0, 1208.36) | 0.86(0.02,53.05) | F | 1.45(0.01,334.42) |
| 8.02 (0.12, 799.81) | 4.19(0.07,214.94) | 2.09 (0.02, 205.9) | 1.13 (0, 800.01) | 0.59(0.01,32.95) | 0.69 (0, 156.5) | G |

A: fresh fecal bacteria; B: frozen fecal bacteria; C: lyophilized fecal bacteria; D: autologous fecal bacteria; E: vancomycin; F: fidaxomicin; G: rectal bacteriotherapy
